# The “CROMa” Project: A Care Pathway for Clinical Management of Patients with Bisphosphonate Exposure

**DOI:** 10.1155/2014/719478

**Published:** 2014-09-22

**Authors:** Mauro Capocci, Umberto Romeo, Fabio Cocco, Isabella Bignozzi, Susanna Annibali, Livia Ottolenghi

**Affiliations:** ^1^Department of Oral and Maxillofacial Sciences, “Sapienza” University of Rome, 6 Caserta Street, 00161 Rome, Italy; ^2^Department of Chemistry and Pharmacy and Department of Surgery, Microsurgery and Medicine Sciences, University of Sassari, Viale S. Pietro, 07100 Sassari, Italy

## Abstract

*Aim.* To describe 7 years of activity of “CROMa” (Coordination of Research on Osteonecrosis of the Jaws) project of “Sapienza” University of Rome. *Materials and Methods.* A preventive and therapeutic care pathway was created for patients with bisphosphonates (BPs) exposure. Demographic, social, behavioural, pharmacological, and clinical variables were registered in a dedicated database. *Results.* In the project, 502 patients, 403 females and 99 males, were observed. Bone pathologies were 79% osteometabolic diseases (OMD) and 21% metastatic cancer (CA). Females were 90% in OMD group and 41% in CA. BP administration was 54% oral, 31% IV, and 11% IM; 89% of BPs were amino-BP and 11% non-amino-BP. Consistently with bone pathology (OMD/CA), alendronate appears to be prevalent for OMD (40% relative), while zoledronate was indicated in 92% of CA patients. Out of 502 cases collected, 28 BRONJ were detected: 17 of them were related to IV BP treatment. Preventive oral assessment was required for 50% of CA patients and by 4% of OMD patients. *Conclusions.* The proposed care pathway protocols for BP exposed patients appeared to be useful to meet treatment and preventive needs, in both oncological and osteometabolic diseases patients. Patients' and physicians' prevention awareness can be the starting point of a multilevel prevention system.

## 1. Introduction 

Recently, an osteonecrosis of the jaws (BRONJ) has been characterized as a main side effect of bisphosphonates (BPs) therapy [1, 2].

This adverse event, first described by Marx and Stern in 2002 [3], has been characterized as nonhealing exposed bone in the mandible or maxilla [4–7] or currently defined as an area of exposed bone in the maxillofacial region that has persisted for more than 8 weeks in a patient on previous or current treatment with a bisphosphonate and without history of radiation therapy to the jaws. Despite this definition, many cases of nonexposed variant of BRONJ have been reported [8].

Mucosal swellings, redness, and purulent exudate sometimes with fistula formation are common. Often the patient complains of pain and discomfort in the mouth, bad taste, and feeding difficulties [9–12]. BRONJ condition may easily progress to severe forms with intractable pain, inability to eat, severe maxillary sinusitis, oroantral fistula, orbital abscess, extraoral fistula, involvement of the lower margin, and fracture of the mandible, especially when it affects debilitated patients [13, 14].

BRONJ has been strongly associated with prolonged use of intravenous (IV) BP (zoledronate and pamidronate) in cancer patients, while patients affected by nonneoplastic diseases and receiving BP with lower dosage or different routes of administration (oral or intramuscular) seem to incur more rarely in this adverse event.

Osteonecrosis is often related to the removal of one or more teeth, to others invasive procedures (i.e., periodontal surgery, dental implant placement, and endodontic surgery), or to local risk factors such as periodontal disease [15], but it can also occur spontaneously, without any apparent dental disease, treatment, or trauma [11].

The cumulative incidence recorded over the years by case-series, case-control, and cohort studies is highly variable, ranging from 0.8 to 12% [2, 16–24].

For patients exposed to IV BP, the rate of spontaneous occurrence is between 0.8 and 1.15%, rising to 6.67%–9.1% when invasive dental procedures are performed. In noncancer patients, the incidence is between 0.01 and 0.04%, increasing from 0.09 to 0.34% in case of dentoalveolar surgery.

Since the first reports focused on BRONJ [1], dental surgical procedures have frequently been described as triggering factors. It is well known that BRONJ can develop with dentoalveolar surgery intervention, and tooth extraction appeared to be the main precipitating risk factor, as it is seen in up to 65% of reported cases [25].

On the other hand, the presence of odontogenic infections exposes patients to considerable risk of BRONJ occurrence. Particularly, cancer patients exposed to IV BP with a history of inflammatory dental disease showed a 7-fold increased risk of developing BRONJ [5]. In fact, many of the cases reported as “spontaneous,” seemingly lacking a triggering factor, may have been the result of a not detected odontogenic focus.

From this point of view, an absolute contraindication to tooth extraction in BP patients may not be advisable. Operative dentistry, endodontics, and periodontal noninvasive treatments remain the first choice to prevent and resolve odontogenic local infections, especially in patients currently or previously treated with BP. Nevertheless “hopeless” nonrestorable teeth should be scheduled for extraction also in patients already exposed to medication, above all when their presence prevents the possibility of proper prosthetic rehabilitation or predisposes to infective conditions.

Furthermore, some inflammatory conditions, such as localized severe chronic periodontitis or extensive periapical lesions from unsuccessful endodontic therapy, not always can be treated by means of elective dental treatments such as periodontal therapy or endodontic retreatment, because they are time-consuming and with uncertain prognosis. Odontogenic infections in subjects scheduled for pharmacological therapy who urgently need to start BP administration for bone malignancies or severe metabolic bone diseases should be effectively and timely addressed, and teeth with poor prognosis or at high risk of infectious complications should be scheduled for extraction.

The aim of the study is to describe 7 years of activity of the “CROMa” (Coordination of Research on Osteonecrosis of the Jaws) project of “Sapienza” University of Rome evaluating the risk variables of patients with past, present, or planned BP exposure, treated with periodontics, oral surgery, and operative dentistry procedures in order to treat or prevent BRONJ, according also to the recent Italian Ministry of Health guidelines of April 2014 [26] and SICMF-SIPMO Italian societies recommendations [27, 28].

## 2. Materials and Methods

### 2.1. The CROMa Project

At the Department of Oral and Maxillofacial Sciences of “Sapienza” University of Rome, in January 2007, a task force of clinicians and researchers set up a Coordination of Research on Osteonecrosis of the Jaws (CROMa). The counselling consists of a multidisciplinary expert group with thorough knowledge of basic and clinical bone biology as well as expertise and daily practice in the fields of preventive dentistry, oral pathology, operative dentistry, and oral and maxillofacial surgery. The aim of CROMa is to prevent or treat established BRONJ and to give relevant pieces of information and advice both to patients and to BP prescribing providers. The task force joins several experts (dentists, oral and maxillofacial surgeons, oral pathologists, oncologists, and an expert in statistics) in order to provide a comprehensive patient-centered oral care delivery.

### 2.2. CROMa Patients Care Pathways

Asymptomatic patients with no signs of osteonecrosis were addressed to the most appropriate dental treatment algorithm, consistently with international protocols, as updated and summarized in Table 2, according to the recent Italian Ministry of Health guidelines [26] and SICMF-SIPMO Italian societies recommendations [27, 28].

All patients, with past, current, or planned BPs therapy, followed 3 possible care pathways.

(A) prevention, (B) surgery, and (C) oral clinics.

Specifically, in the (A) path, patients received professional oral hygiene and personal oral hygiene instructions; in the (B) path, they received surgical care: dental extractions and/or surgical treatment of BRONJ were performed; hopeless teeth, being potential or actual infection sites, were treated with extractions. In the (C) path, patients were treated with operative dentistry and/or endodontics and/or periodontal treatments, supported also by various types of laser (analgesic or biostimulating low level laser therapy, surgical lasers for soft tissues, and ablative lasers for bone treatment) in order to remove or prevent odontogenic infections and/or to relief pain [29].

Patients could follow combinations of the care pathways, according to treatment needs.

All the established BRONJ were treated combining (B) and (C) pathways, in order to give necessary surgical (traditional and/or laser guided surgery) and/or biostimulating (low level laser therapy) and/or medical treatments (antibiotics, analgesics, antibacterial rinses, integrators of the immune system, etc.). All patients exposed to BP underwent clinical procedures according to international guidelines.

### 2.3. Diagnostic Protocol

Oral health status was assessed and the presence of jaws pathological or anatomical conditions, acting as potential BRONJ risk factors, or the finding of suspected osteonecrosis was recorded through physical examination.

For all patients, to exclude the presence of BRONJ, in addition to anamnestic notes and clinical features, laboratory tests and radiographic data, such as orthopantomographs and full periodontal radiographic exams, were harvested and examined. No bone turnover biomarkers were used, as they were judged to be not completely reliable in predicting risk [30]. In case of suspected BRONJ, to confirm diagnosis, computed tomography (CT) scans imaging and further laboratory tests were requested, as needed. Lesions were staged in the beginning according to AAOMS Position Paper 2007 [4], modified in 2009 [5]. Later, we used SIPMO/SICMF recommendations 2011 [27] and 2013 [28] (Table 1). Every new classification we adopted through these 7 years of activity has been followed by a review of our BRONJ patient collected data (radiographies, clinical chart, pictures, etc.) to make every case up to date.

### 2.4. Data Collection

A unified clinical chart was developed in order to collect all necessary data in a digital online database.

Age, gender, presence of systemic diseases, use of any drugs, and the main systemic and local risk factors were registered. Patients were asked for a comprehensive history concerning the use, dose, frequency, and duration of therapy with BP.

The parameters to define a patient at higher or lower risk to develop BRONJ were identified in the limit of 3 years for oral and IM BP therapy and of 8 infusions for IV BPs [24].

Only patients with past, present, or planned BP exposure were included in the CROMa project, with or without established BRONJ.

Patients have been catalogued following a chronological sequence into a Microsoft Access database, editable and searchable online by all the main components of the CROMa project.

### 2.5. Data Analysis

The collected samples (January 2007–March 2014) of patients were examined according to gender (male/female), age, bone disease (osteometabolic (OMD)/oncological (CA)), type of drug (amino-BP/non-amino-BP), BP active ingredient (alendronate/zoledronate, etc.), the route of administration (oral (OS)/intramuscular (IM)/intravenous (IV), or their combination), administration time (months of therapy, then divided into 2 categories for OS/IM (< or >3 years) and 2 categories for IV (< or >8 infusions)), and the timing (current, past, or planned BP therapy).

In addition, systemic and local risk factors for BRONJ and BRONJ presence and staging were also analyzed.

Data were coded and imputed into an Excel 2013 spreadsheet (Microsoft Inc., Redmond, WA, USA) and checked to verify the accuracy. Statistical analysis was performed using Stata 13.0 (San Diego, CA, USA) for the Macintosh operating system. Initially, univariate analyses were performed on the clinical condition parameters and potential risk indicators to describe the variables and distributions. Then a descriptive statistical analysis was performed. To avoid the attenuating effect of unequal variability among groups on the value of *t*, a square root transformation was performed when the response variable was a count. The association between BRONJ and background factors was tested using the *χ* ^2^ test.

A stepwise logistic regression model was built using the presence of at least one BRONJ lesion as the dependent variable. Gender has been identified as a modifier effect in the statistical analysis. Therefore, two different logistic models stratified by gender were run following robust statistics (24. Wilcox RR. Introduction to Robust Estimation and Hypothesis Testing (Third Edition) Elsevier Inc. 2013). Unless stated otherwise, the criterion for statistical significance was set at *α* = .05.

## 3. Results

From January 2007 to March 2014, 502 patients (Table 3) were included in the CROMa project, including 403 females and 99 males aged between 8 and 90 years. Bone diseases were 79% of osteometabolic type (OMD, 398 cases, of which 310 were for osteoporosis (78% rel. ∣ 62% tot.), 54 for osteogenesis imperfecta (13% rel. ∣ 11% tot.), and 13 for osteopenia (3% rel. ∣ 2,5% tot.)) and 21% of oncological type (CA, 104 cases, including 34 for bone metastases from prostate cancer (33% rel. ∣ 7% tot.), 28 from mammary cancer (27% rel. ∣ 5% tot.), and 14 from multiple myeloma (13% rel. ∣ 3% tot.)).

The OMD concerned 90% of women and 10% of men, while CA patients were 41% females and 59% males. The routes of BP administration were mostly oral (54%), followed then by IV therapies (31%), IM (11%), and an association of these in 3% of cases.

The active principles administered have seen in the whole group a prevalence of amino-BP drugs (89%), including alendronate (33% tot.), zoledronic acid (21% tot.), risedronate (17% tot.), neridronate (12% tot.), and ibandronate (6% tot.), compared to non-amino-BP administration (11%) represented only by clodronate.

The distribution according to bone diseases (OMD/CA) has seen alendronate as a drug of choice for OMD (40% rel.) followed by risedronate (21% rel.), while, in the other category, zoledronic acid was indicated in 92% of patients with metastatic bone cancer.

An analysis of the BP planned therapies group highlights that, out of 155 cases of IV therapy, 78 patients (50%) were referred for oral health assessment before starting the drug administration: the trend is completely different for the oral therapies (4%, 12 cases out of 270) and IM therapies (3%, 2 cases out of 60).

Out of 502 patients (Table 4), 28 differently staged BRONJ were intercepted at first examination (3 at Stage 0, 8 at Stage 1, 12 at Stage 2, and 5 at Stage 3), 17 in the CA group (16,4%), and 11 in the OMD group (2,2%). The outcome is overlapping with the therapy regimen variable (17 from IV BP administration (11% of all IV) and 11 from oral BP drugs (4,1% of all OS)). No BRONJ in our study has been related to exposition to non-amino-BP. From the logistic regression model (Table 5), we can observe how BRONJ risk in male patients is significantly connected principally to therapy intervals, while in women the risk is influenced also by behavioral habits, oncologic type of bone disease, and therapy regimen.

Between 28 BRONJ patients, 13 were being treated with chemotherapy, 8 were receiving prolonged therapy with glucocorticoids, 4 were smokers, and 2 had diabetes.

In addition, clinical and radiological examination underlined that 6 of them had odontogenic infections, and the same percentage had poor oral hygiene and periodontal disease.

## 4. Discussion

The CROMa project was created with the primary intent to be a benchmark for dental patients exposed to the BP drugs or about to take them. Meticulous collection of personal, epidemiological, and clinical data has provided a fairly complete overview of the population exposed to the drug who presented to our department. Interestingly, 86% of the patients with nonintravenous BP therapy were addressed to the Department of Dentistry for a routine dental visit or for emergency dental treatments; only 4% was asked for an oral health assessment before BP administration. Overall, these patients showed poor awareness of the clinical concerns associated with BP intake, and poor information had been provided by the prescribing physician about the possibility of BRONJ occurrence after dentoalveolar surgical procedures.

On the contrary, patients with intravenous BP therapy for bone malignancies or dysplastic bone diseases showed a greater awareness and understanding of the issue and were referred to CROMa by the specialist who treated them for the underlying disease (50% were referred before IV BP therapy).

This disparity is probably due to the statistics which define a higher risk only or above all for the IV therapies. Nevertheless, as shown by our data, the BRONJ occurrence subsequently to oral BP administration is possible, also considering the much higher number of patients exposed to oral BP administration than to the IV one. In 2005, alendronate was the 15th most prescribed drug with approximately 18 million prescriptions and risedronate was the 37th with almost 10 million prescriptions [31]. Furthermore, our study shows how BPs are prescribed even in case of osteopenia (2,5% tot.) rather than prescribing other drugs with fewer possible side effects.

Overall, data analysis shows that the most at-risk situation is in the metastatic bone cancer group, treated with IV administration of NBP, for a long therapy interval (more than 8 infusions), with females being much more represented in the OMD group, due to postmenopausal osteoporosis.

At the state of knowledge, a specific evidence-based treatment protocol for BRONJ has not been established. At present, literature provides clinician with only a few indications of possible treatment algorithms through case reports and case series. In 2013, a new clinical-radiological staging was defined, which considers also the radiographic extension of BRONJ and the further classification of Stage 1 and Stage 2 in asymptomatic (1A) and symptomatic (1B) [28].

However, as all current treatments appear to be suboptimal and no consensus has been reached on completely effective and predictable approach once BRONJ has developed, the best chances are in prevention.

The most important goal of CROMa project is specifically prevention. Currently, preventive approach is not yet common among prescribers of oral BP. Prevention should be more strongly promoted by sharing knowledge in the involved medical community and establishing a fruitful cooperation with the specialist prescriber of the BP drug, working as a team on behalf of patient.

Moreover, in our study, all the patients with BRONJ who have been treated with surgery following our protocols and algorithms have reported a relief of the symptoms and an improvement of their quality of life. No recurrence of BRONJ has been reported during the follow-ups after 4, 8, and 12 months from surgery. Furthermore, no evidence of BRONJ has been found in any OMD or CA patient during the following planned scaling/root planning treatments.

## 5. Conclusions

Although BRONJ is a relatively rare side effect of BP therapy, it is still an important issue for the medical community due to the severity of the condition and the lack of a thorough understanding of the pathophysiology and predisposing risk factors. An accurate delineation of the pathogenic mechanisms at the cellular and biochemical levels, as well as clinical and laboratory markers for prediction of BRONJ susceptibility in the single subject, is still lacking. From a clinical point of view, no evidence-based recommendations exist about the dental treatments that can be performed without risk or with appropriate risk-benefit ratio. Furthermore, the protocols of treatment to manage overt disease appear to be suboptimal.

The preventive and therapeutic protocols of BRONJ currently proposed appeared to be useful.

Our patients, referred by other specialists or simply intercepted during the medical history collection in the first observation unit, have been treated in order to meet their immediate needs and then to minimize BP-related risks for oral health, following the best practice preventive and treatment protocols.

Focusing on prevention, it is important that the involved medical community share knowledge and that the physicians take a conscious attitude so as to provide patients with the highest quality of oral health care, before starting BP therapy, in order to improve the care and oral health-related quality of life of patients, in both oncological and osteometabolic diseases. Prevention awareness, aided also by the networking use of an online database, can be the starting point of a multilevel prevention system.

## Figures and Tables

**Table 1:** 2013 SIPMO/SIMCF clinical-radiological staging of BRONJ [28].

Stage 1	**Focal BRONJ**: in the presence of at least 1 minor clinical sign or of ***bone thickening on CT limited to mandibular or maxillary dentoalveolar process***∗, with or without other early radiological signs.
	**Minor clinical signs and symptoms**: halitosis, odontogenic abscess, mandibular asymmetry, pain of dental and/or boned origin, bone exposure, mucosal fistula, postextractive mucosal healing failure, rapid onset tooth mobility, paresthesia/dysesthesia of the lips, purulent leakage, spontaneous seizure of bone fragments, trismus, and soft tissues swelling.
	**Signs on CT**: ***trabecular thickening, bone marrow focal osteosclerosis***, with or without thickening of the alveolar crest, postextractive socket persistence, and periodontal space flare.
	**(A) Asymptomatic.**
	**(B) Symptomatic** (presence of pain and/or suppuration).

Stage 2	**Widespread BRONJ**: in the presence of at least 1 minor clinical sign or of ***bone thickening on CT, also extended to the mandibular or maxillary basal process***, with or without late radiological signs.
	**Minor clinical signs and symptoms**: as for Stage 1.
	**CT signs**: ***widespread osteosclerosis***, with or without oroantral and oronasal fistula, thickening of the inferior alveolar nerve canal, periosteal reaction, bone seizure, and sinusitis.
	**(A) Asymptomatic.**
	**(B) Symptomatic** (presence of pain and/or suppuration).

Stage 3	**Complicated BRONJ: **as in Stage 2, in the presence of 1 or more of the following signs:
	**Minor clinical signs:** extraoral fistula, leakage of fluid from the nose, and preternatural mobility of the jaw with or without occlusion preservation.
	**CT signs: **mucocutaneous fistula, pathologic fracture, osteolysis extended to maxillary sinus, and cheekbone and/or hard palate osteosclerosis.

*Dentoalveolar bone anatomical structure that constitutes the skeletal support for the teeth. By definition, the dentoalveolar process ends in craniocaudal direction immediately below the root of the teeth.

**Table 2:** Oral procedures in patients with current/past or planned BP therapy [28].

Malignancies				Osteometabolic disorders	
Treatment		Planned BF therapy	Current/past BF therapy	Planned or <3 years of NBP therapy	>3 years of NBP therapy or <3 years with risk factors for BRONJ
Dentoalveolar surgery	Extractive procedures	*Recommended *	*Recommended *	*Recommended *	*Recommended *
		Simple extraction^1^	Surgical extraction^2^	Simple extraction	Surgical extraction^2^
		*To wait* until mucosal healing before starting BF therapy (4–6 weeks)	*Recommended therapy suspension* from extraction day until mucosal healing (4–6 weeks)	—	—
	Preimplant surgery	Not recommended	Not recommended	Possible	Possible^4^

Implantology		Not recommended	Not recommended	Possible^3^	Possible^3,4^

Periodontal surgery	Therapeutic	Recommended^2,5^	Recommended^2,5^	Recommended	Recommended^2^
		*To wait* until mucosal healing before starting BF therapy	*Recommended therapy suspension *	—	—
		(4–6 weeks)	from extraction day until mucosal healing		
			(4–6 weeks)		
	Elective	Not recommended	Not recommended	Possible	Possible

Endodontic surgery		Recommended^2,5^	Recommended^2,5^	Recommended	Recommended^2^

Periodontal therapy (scaling/root planning)		Recommended	Recommended	Recommended	Recommended
			(every 4 months)		(every 4–6 months)

Conservative		Recommended	Recommended	Recommended	Recommended

Endodontics		Recommended	Recommended	Recommended	Recommended

Orthodontics		Possible	Possible (recommended low orthodontic forces)	Possible	Possible

Fixed prosthesis		Possible	Possible^6^	Possible	Possible^6^

Removable prosthesis		Possible	Possible	Possible	Possible
			*Avoid* injuries and pressure sores, to use soft liners eventually		*Avoid* injuries and pressure sores, to use soft liners eventually
			(control of the prosthesis every 4 months)		(control of the prosthesis every 4–6 months)

^1^If BP therapy cannot be delayed, choose surgical extraction; ^2^use mucoperiosteal flap for primary closure of the surgical site; ^3^informed consent for not defined long-term BRONJ risk; ^4^informed consent for not defined short-term BRONJ risk; ^5^only for the treatment of significant infectious-inflammatory processes, not otherwise controllable using noninvasive methods; ^6^respect of the biological width (control of cervical closure-possible supragingival closure).

**Table 3:** Data from CROMa database.

CROMa patients	502	
	Males	99
	Females	403
	Age	8–90
	Paediatric	11%
	Adults	89%

		Number

Osteometabolic diseases (OMD)	79% (398)	
	Postmenopausal osteoporosis	310
	Osteogenesis imperfecta	54
	Osteopenia	13
	Osteoarthritis	7
	Secondary osteoporosis	6
	Glucocorticoid-induced osteoporosis	3
	Fibrous dysplasia	2
	Paget's disease	1
	Other	2

		Number

Metastatic cancer (CA)	21% (104)	
	Prostate cancer bone metastasis	34
	Mammary cancer bone metastasis	28
	Multiple myeloma	14
	Renal cancer bone metastasis	11
	Pulmonary cancer bone metastasis	9
	Other	8

BP administration	BP therapy	58 (11%)
	NBP therapy	444 (89%)
	OS	54%
	IV	31%
	IM	11%
	Association	3%

Patients with no BRONJ		474 (94,42%)
Patients with BRONJ		28 (5,58%)
BRONJ from oral BP		11
BRONJ from IV BP		17

**Table 4:** Sample distribution of CROMa patients by BRONJ presence.

	BRONJ (*n* %)∗	Healthy (*n* %)∗	OR (95% CI)
Osteometabolic disease	11 (2,2%)	387 (97,8%)	—
Metastatic cancer	17 (16,4%)	87 (83,6%)	0.20 (0.11–0.33)

			χ^2^ for trend 28.82, P < .01

Therapy intervals			
No therapy	1 (1%)	92 (99%)	—
<3 years	7 (3,9%)	172 (96,1%)	0.02 (0.01–0.07)
>3 years	4 (2,7%)	146 (97,3%)	0.03 (0.01–0.07)
IV < 8 infusions	2 (11,8%)	15 (88,2%)	0.13 (0.03–0.58)
IV > 8 infusions	14 (22,2%)	49 (77,8%)	0.29 (0.16–0.52)

			χ^2^ for trend 41.23, P < .01

Therapy regimen			
Association between methods	0 (0%)	17 (100%)	—
IV	17 (11%)	138 (89%)	0.12 (0.07–0.20)
IM	0 (0%)	60 (100%)	
OS	11 (4,1%)	259 (95,9%)	0.05 (0.02–0.07)

			χ^2^ for trend 4.31, P = .04

*The percentage (*n* %) is not absolute but is relative to the specific field.

**Table:** (a) Male

Variable	OR	Robust (SE)	*P*	95% CI
Behavioral habits	.92	.15	0.62	1.07–1.39
Therapy intervals	3.14	1.01	<.01	1.68–5.89

Number of observations= 61; log likelihood= −13.27; *χ* ^2^_(2)_ = 24.50; *P* value < .01.

**Table:** (b) Female

Variable	OR	Robust (SE)	*P*	95% CI
Behavioral habits	1.22	.08	<.01	1.07–1.39
Oncology bone disease	17.90	14.03	<.01	3.85–83.25
Therapy regimen	2.85	1.41	.03	1.08–7.50
Therapy intervals	2.37	.73	<.01	1.29–4.32

Number of observations= 403; log likelihood= −60.03; *χ* ^2^_(4)_ = 33.10; *P* value < .01.
